# Patient-specific proximal femoral plate combined with a modular 3D-printed osteotomy guide system in children with developmental dysplasia of the hip: a retrospective comparative study

**DOI:** 10.3389/fpubh.2026.1891091

**Published:** 2026-07-31

**Authors:** Haoran Qi, Pengkun Wang, Feng Lian

**Affiliations:** 1 Department of Orthopedic Surgery, The Fourth Affiliated Hospital of Harbin Medical University, Harbin, Heilongjiang, China; 2 Center for Leading Medicine and Advanced Technologies of IHM, The First Affiliated Hospital of USTC, Division of Life Sciences and Medicine, University of Science and Technology of China, Hefei, Anhui, China

**Keywords:** 3D printing, developmental dysplasia of the hip, modular osteotomy guide, patient-specific plate, pediatric hip surgery, proximal femoral osteotomy

## Abstract

**Background:**

To evaluate the clinical value of a patient-specific proximal femoral plate combined with a modular 3D-printed osteotomy guide system in children with developmental dysplasia of the hip (DDH) undergoing proximal femoral osteotomy.

**Materials and methods:**

This retrospective study included children with DDH who underwent proximal femoral osteotomy assisted by a modular 3D-printed osteotomy guide system. Patients were divided into a customized plate group and a conventional locking compression pediatric hip plate (LCP-PHP) group. Perioperative outcomes, including blood loss, operative time for the femoral procedure, and fluoroscopy frequency, were compared. Radiographic parameters, including neck-shaft angle (NSA), femoral anteversion angle (FAA), acetabular index (AI), and center-edge (CE) angle, were assessed postoperatively and at final follow-up. Functional outcomes, complications, and measurement reliability were also evaluated.

**Results:**

A total of 40 patients were included. The mean follow-up duration was 32.32 ± 4.18 months in the customized plate group and 31.39 ± 3.88 months in the conventional LCP-PHP group. No major complications were observed in either group. Compared with the conventional LCP-PHP group, the customized plate group showed a significantly smaller loss of planned NSA correction, whereas FAA correction loss was similar between groups. No significant between-group differences were found in blood loss, operative time, fluoroscopy frequency, postoperative radiographic parameters, final follow-up outcomes, or functional results. Reliability analysis demonstrated excellent reproducibility of radiographic measurements, supporting the validity of the difference in NSA correction loss.

**Conclusions:**

A patient-specific proximal femoral plate combined with a modular 3D-printed osteotomy guide system appears to be a feasible and safe option for proximal femoral osteotomy in children with DDH. Compared with the conventional LCP-PHP, the customized plate more accurately maintained the planned NSA correction and may help improve correction accuracy in pediatric hip reconstruction.

## 1 Introduction

Developmental dysplasia of the hip (DDH) is one of the most common pediatric orthopedic disorders and may result in persistent hip instability, abnormal development of the acetabulum and proximal femur, and early degenerative joint changes if left untreated (1–3). In children with more severe deformities, particularly those presenting with increased femoral anteversion, coxa valga, or irreducible hip dislocation, surgical intervention is often required to restore hip congruency and stability (4, 5). Among the available surgical procedures, proximal femoral osteotomy is commonly used in the management of DDH because it allows femoral shortening, derotation, and varus correction (6). These adjustments help reduce soft-tissue tension, facilitate stable concentric reduction, improve proximal femoral alignment, and thereby promote subsequent acetabular remodeling (7, 8).

**Figure 1:**
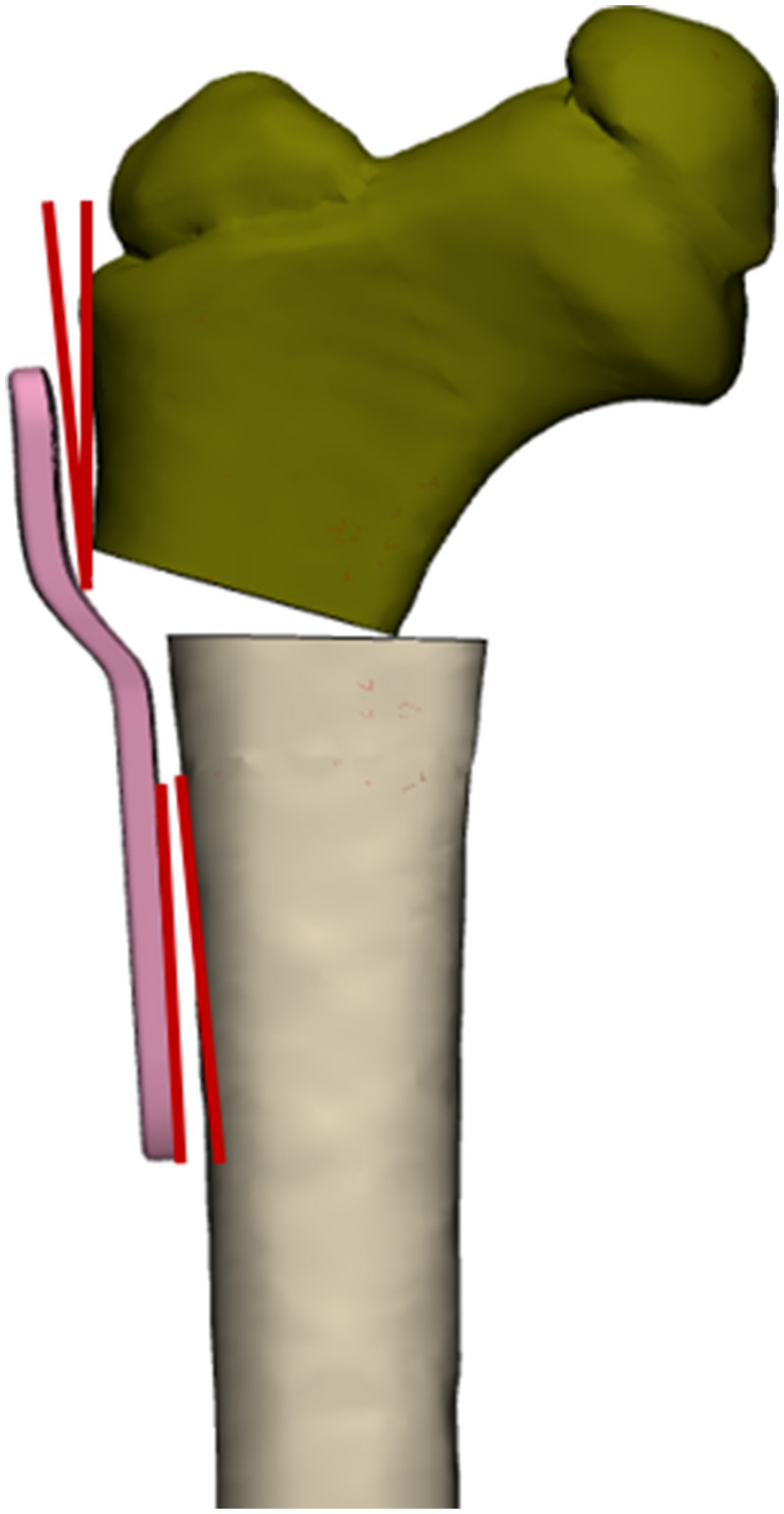
Schematic illustration of residual mismatch between the conventional LCP-PHP and the corrected proximal femoral anatomy after osteotomy. The pink structure represents the conventional LCP-PHP. Residual mismatch can be observed between both the proximal and distal plate segments and the corrected femoral surface. LCP-PHP: locking compression pediatric hip plate.

For fixation following proximal femoral osteotomy, the locking compression pediatric hip plate (LCP-PHP) is a commonly used pre-contoured implant that provides relatively stable fixation after varus-producing and derotational osteotomy in children (9–11). However, in our previous studies and clinical application, we also observed that the conventional LCP-PHP did not always provide satisfactory conformity to the corrected proximal femoral anatomy after osteotomy (Figure 1). This observation suggested that, as osteotomy accuracy improves, the fixation implant itself may become another key factor influencing maintenance of the planned correction. Given the substantial interpatient variation in proximal femoral morphology, a pre-contoured off-the-shelf implant may not adequately match the individualized postoperative anatomy after derotation and varus correction (12). Therefore, improving plate–bone conformity may be necessary to better maintain the planned correction after guide-assisted osteotomy.

In recent years, digital orthopedic technologies, including three-dimensional (3D) reconstruction, virtual simulation, and 3D printing, have been applied in the surgical management of DDH (13, 14). These technologies have improved the precision of preoperative planning and facilitated the development of patient-specific surgical assistance tools. In our previous work, we developed a novel modular 3D-printed osteotomy guide system for proximal femoral osteotomy in children with DDH (15, 16). By dividing the conventional integrated guide into several modular components, this system improved intraoperative flexibility and allowed the preoperative three-dimensional correction plan to be implemented through relatively limited surgical exposure. Our previous studies showed that guide-assisted proximal femoral osteotomy simplified the surgical procedure, shortened operative time, reduced intraoperative blood loss, and decreased radiation exposure, while achieving satisfactory radiographic and functional outcomes. On this basis, the present study further incorporated patient-specific plate design into the preoperative digital workflow in an attempt to address the problem of insufficient conformity between the conventional LCP-PHP and the corrected proximal femoral morphology.

Based on CT-derived three-dimensional reconstruction and virtual osteotomy simulation, we designed a customized proximal femoral plate that matched the predicted femoral anatomy after derotation and varus correction. By optimizing the contour of a standard pediatric hip plate according to the simulated post-osteotomy bone surface, the customized plate was intended to improve conformity to both the proximal and distal osteotomy segments. This design was expected to be particularly suitable for the face-to-face osteotomy configuration created by our modular guide system, thereby facilitating more accurate fixation and reducing intraoperative adjustment.

Therefore, the aim of this study was to compare the perioperative performance, correction accuracy, and short-term clinical outcomes of a customized proximal femoral plate and a conventional LCP-PHP in children with DDH undergoing proximal femoral osteotomy assisted by a modular 3D-printed guide system. We hypothesized that, compared with the conventional plate, the customized plate would provide better conformity to the post-osteotomy femoral anatomy and more accurately maintain the planned correction.

## 2 Materials and methods

### 2.1 Patients

This retrospective study included consecutive children aged 18 months to 6 years with Tönnis type II–IV DDH who underwent surgery assisted by a novel modular 3D-printed osteotomy guide system at our institution between September 2020 and February 2023. According to the type of internal fixation used, the patients were divided into two groups: a conventional group (18 cases) treated with a standard LCP-PHP and a customized group (22 cases) treated with a patient-specific proximal femoral plate.

The inclusion criteria were as follows: (1) unilateral DDH requiring open reduction, Salter pelvic osteotomy, and proximal femoral varus-derotational osteotomy based on preoperative assessment of proximal femoral morphology and hip stability; (2) treatment performed with assistance from the novel modular 3D-printed osteotomy guide system; (3) complete clinical and imaging records; (4) no history of other hip disorders; (5) no obvious lower-limb deformities; and (6) follow-up duration greater than 12 months. The exclusion criteria were as follows: (1) bilateral DDH; (2) previous ipsilateral hip surgery or residual deformity after prior treatment; (3) revision surgery during the follow-up period; (4) hip dislocation secondary to other causes; and (5) incomplete medical records.

This study was conducted in accordance with the Declaration of Helsinki. The study protocol was approved by the Ethics Committee of the Fourth Affiliated Hospital of Harbin Medical University (approval No. 2025-LLSC-03). The requirement for informed consent was waived because of the retrospective study design.

### 2.2 Design and fabrication of customized plates

**Figure 2:**
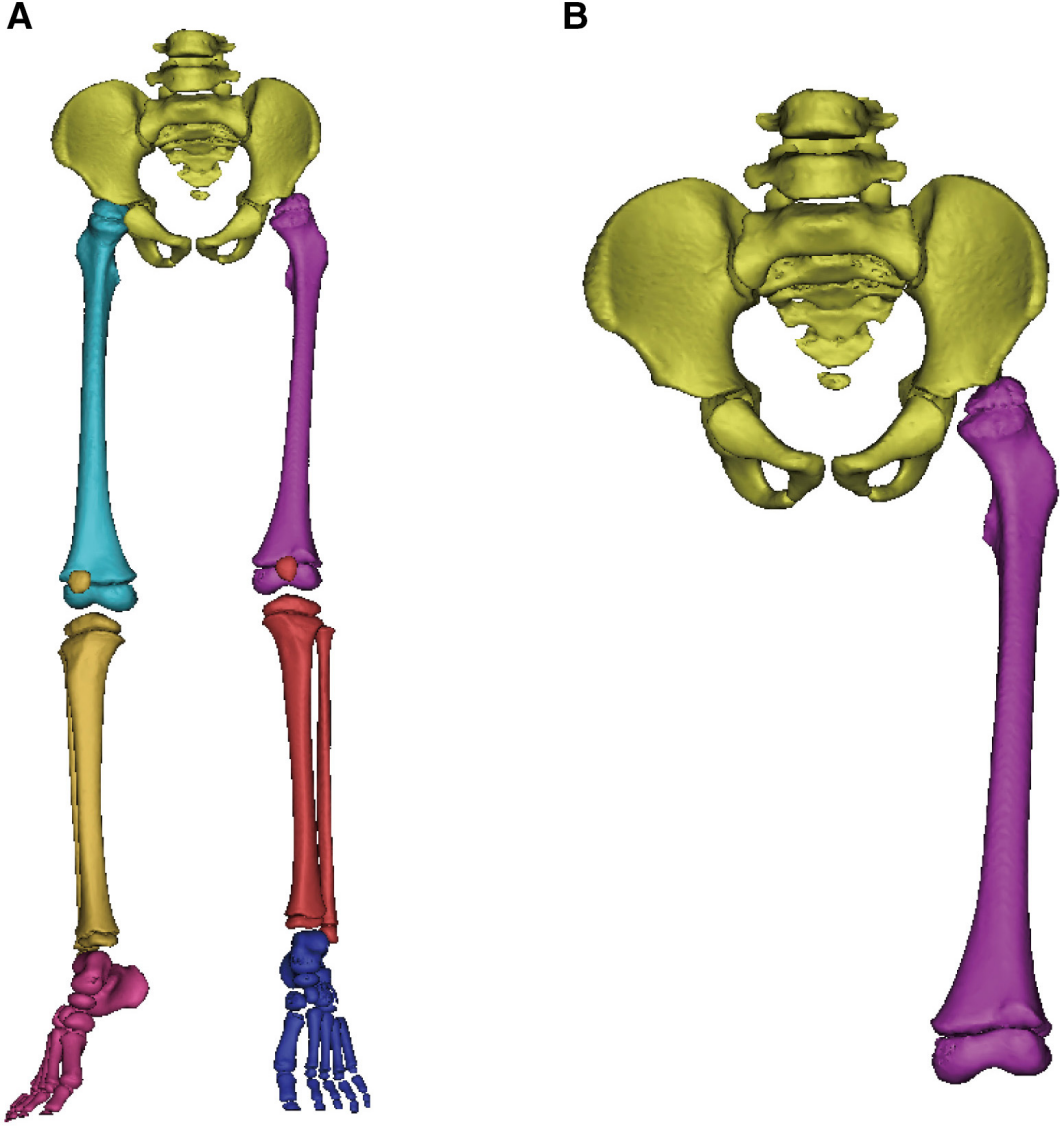
**(A)** The patient's preoperative CT data. **(B)** The CT data were imported into Mimics for three-dimensional reconstruction.

**Figure 3:**
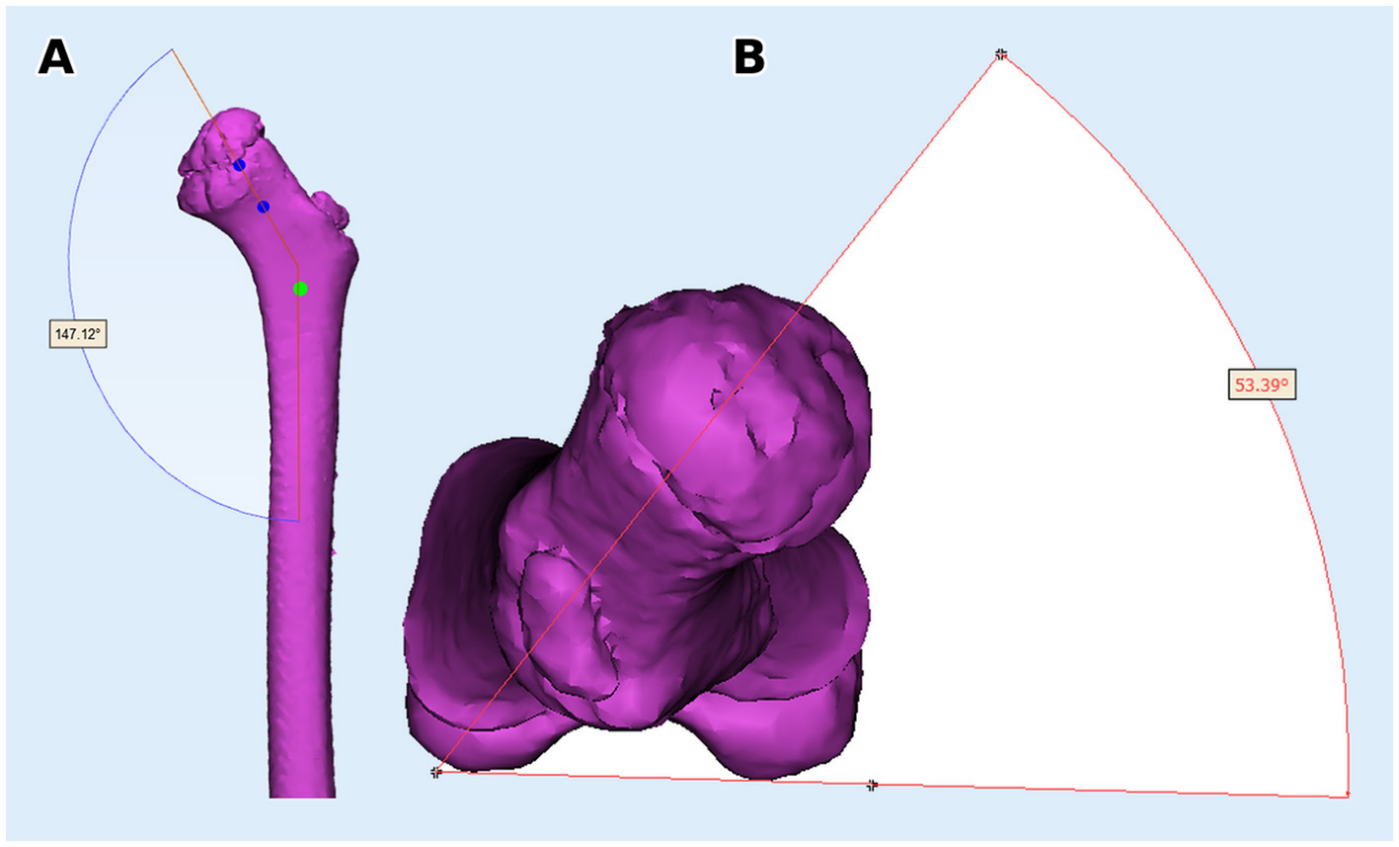
**(A)** Measurement of the NSA of the femur. **(B)** Measurement of the FAA. NSA, neck-shaft angle; FAA, femoral anteversion angle.

Preoperative CT data of the affected hip and proximal femur were acquired for three-dimensional reconstruction and digital surgical planning. Scanning parameters were adjusted according to patient age and body size, and the scanning range was limited to the anatomical region required for reconstruction. The CT data were imported into Mimics 21.0 software (Materialize, Leuven, Belgium) in DICOM format. Bone segmentation and image processing were then performed to generate a three-dimensional model of the proximal femur. The femoral model was subsequently refined, and relevant anatomical structures were separated and color-coded as needed to facilitate visualization of the osseous anatomy (Figure 2). In Mimics 21.0, the femoral anteversion angle (FAA) was measured according to the reconstructed femoral anatomy (Figure 3B).

The reconstructed model was then imported into 3-matic 13.0 software (Materialize, Leuven, Belgium), where the neck-shaft angle (NSA) was measured as the angle between the femoral neck axis and the femoral shaft axis within the plane defined by the neck and shaft (Figure 3A). Based on these measurements, the required derotation and varus correction angles were determined preoperatively.

**Figure 4:**
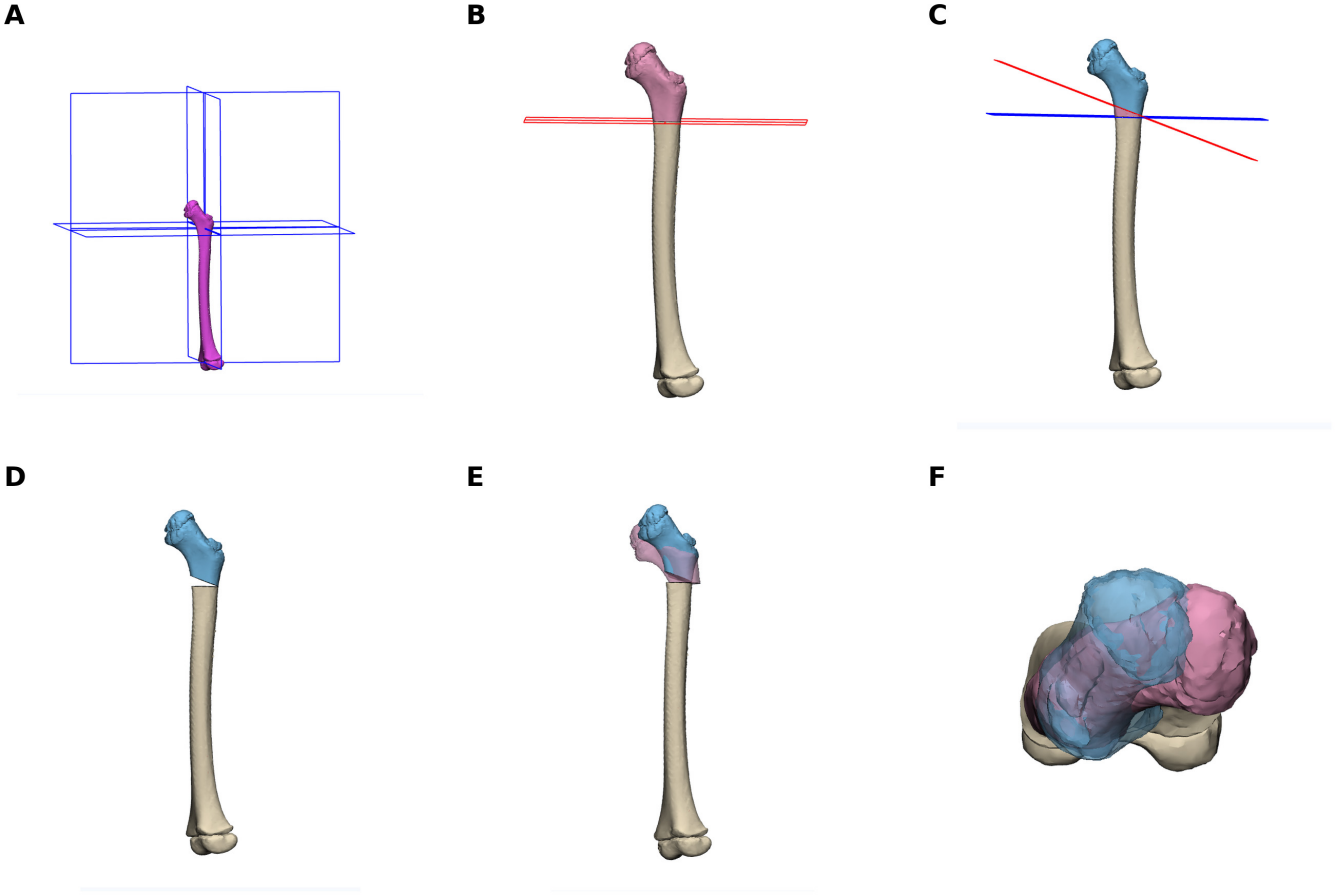
**(A)** The coronal, sagittal, and transverse planes were established in 3-matic. **(B)** A transverse osteotomy was performed. **(C)** An oblique osteotomy was performed. **(D)** The triangular bone fragment was removed. **(E)** The NSA was adjusted, and the osteotomy surfaces were brought into face-to-face contact. **(F)** The FAA was adjusted. NSA: neck-shaft angle, FAA: femoral anteversion angle.

**Figure 5:**
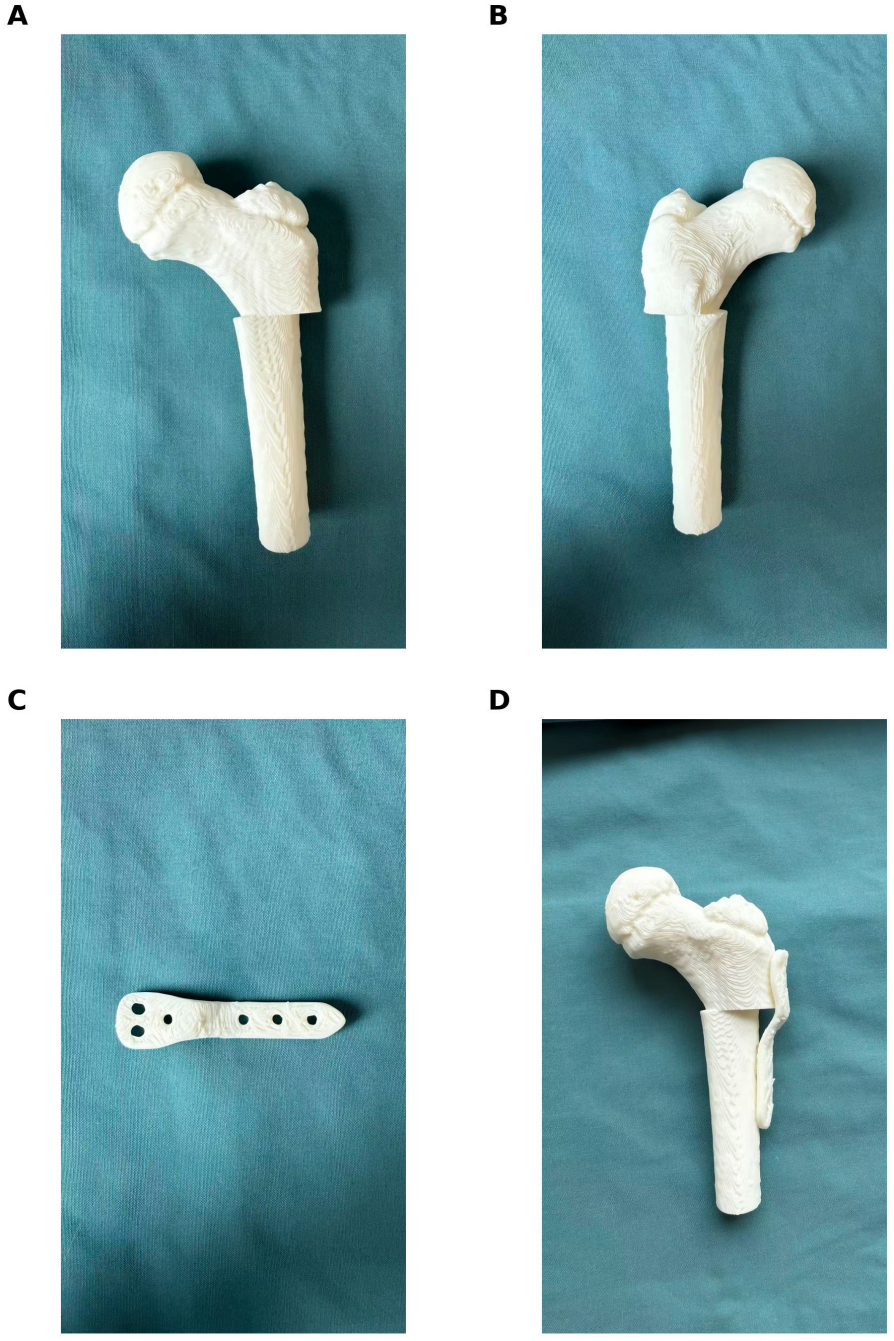
**(A)** Anterior view of the 3D-printed model of the proximal femur after simulated osteotomy. **(B)** Posterior view of the 3D-printed model of the proximal femur after simulated osteotomy. **(C)** 3D-printed model of the customized plate. **(D)** Fit between the proximal femoral model and the customized plate.

Subsequently, virtual osteotomy simulation was performed in combination with the predesigned modular guide system, and the proximal femur was adjusted in three dimensions to obtain the desired postoperative morphology (Figure 4). After completion of the virtual osteotomy and correction, the model data were exported in STL format and imported into FashPrint 5 software (FlashForge, China). A physical femoral model was then fabricated using a 3D printer for preoperative planning and patient-specific plate design (Figures 5A, B).

Based on the three-dimensional model, orthopedic surgeons collaborated with biomedical engineers to design a patient-specific plate. The virtually corrected femoral model was imported into Unigraphics NX software (Siemens PLM Software, USA), where the position, length, curvature, and conformity of the plate were designed according to the patient's proximal femoral anatomy and the corrected morphology. The number, arrangement, and orientation of the screw holes were also determined during this process.

**Figure 6:**
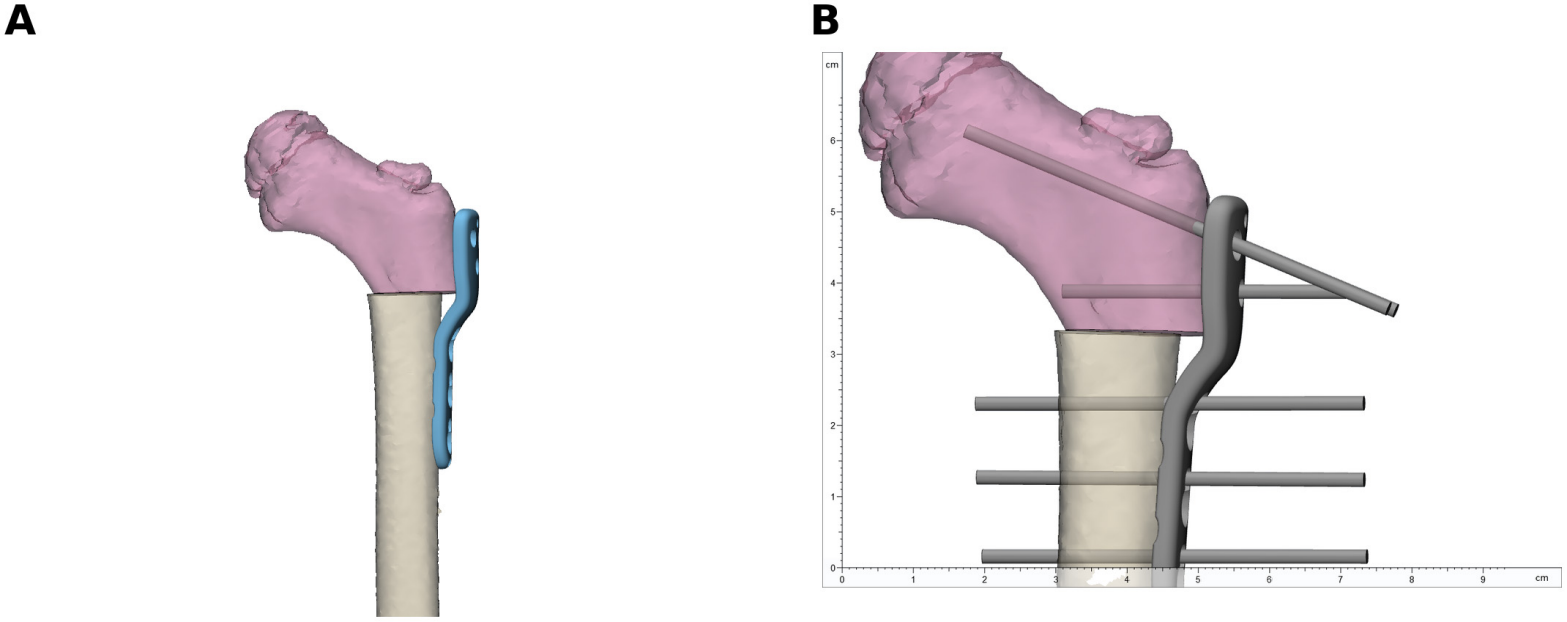
**(A)** Fit between the customized plate designed in Mimics and the three-dimensional femoral model. **(B)** Design of the customized plate, including determination of the optimal plate position and screw trajectories. The femoral model was rendered transparent to verify that the screws did not penetrate the joint cavity, and the lengths of the fixation screws were measured.

After the preliminary design was completed, a physical prototype of the plate was printed using PLA material with FashPrint 5 software (Figure 5C). The plate model was then reverse scanned and imported back into Mimics software to verify its conformity with the three-dimensional femoral model (Figure 6A). By rendering the bone model transparent, the trajectory and orientation of the screws could be clearly visualized to ensure that the screws would not penetrate the joint cavity or other critical anatomical structures. The screw lengths were determined using the measurement tools within the software, and the recommended screw lengths were labeled in the plate design to guide intraoperative implantation (Figure 6B).

**Figure 7:**
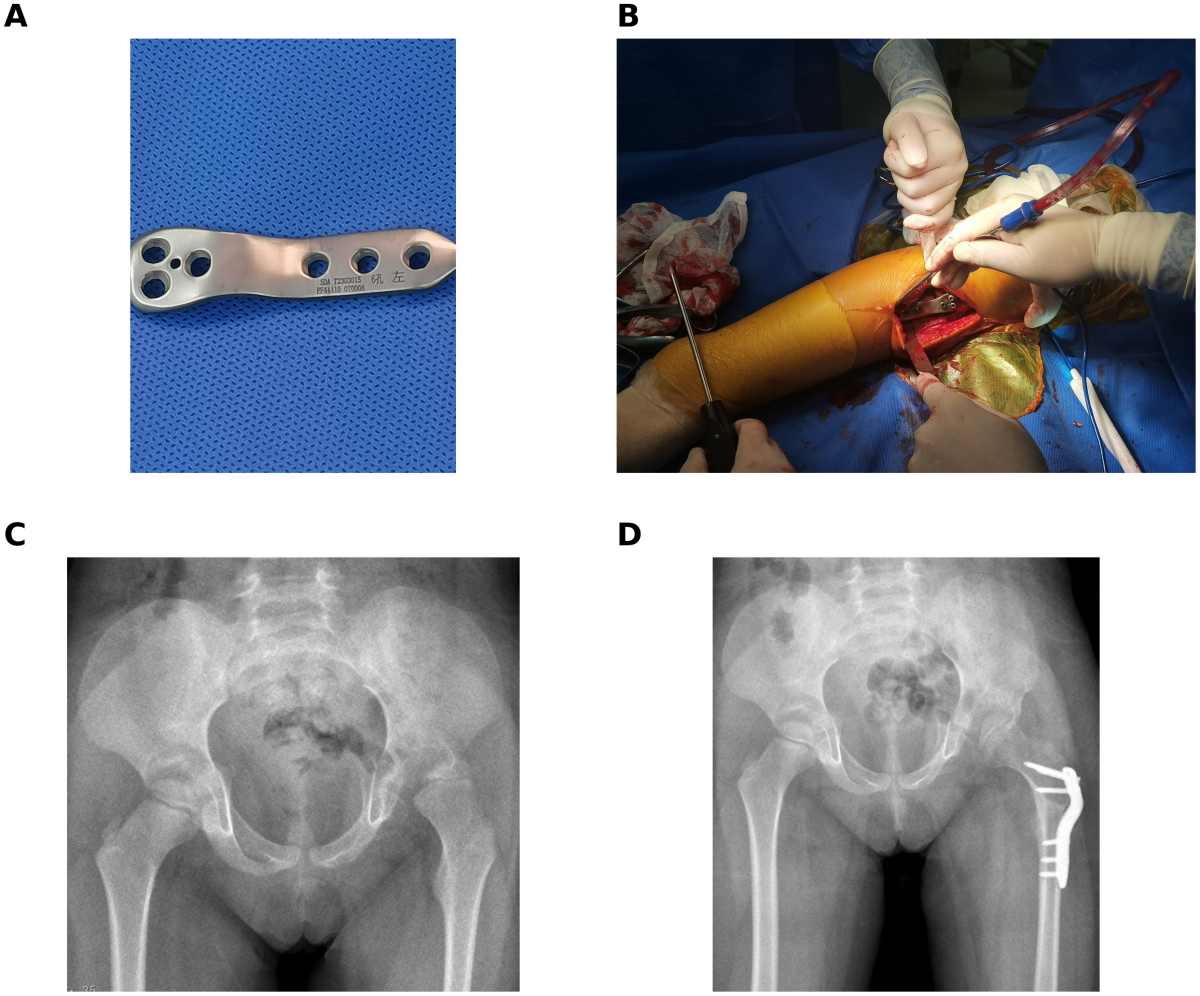
**(A)** Gross appearance of the customized plate. **(B)** Intraoperative use of the customized plate. **(C)** Preoperative X-ray of the patient. **(D)** Postoperative X-ray of the patient.

Finally, based on the optimized design, the customized plate was manufactured from pure titanium TA3 in a professional medical device manufacturing facility (Figure 7). After fabrication, the plate underwent strict quality inspection and sterilization before being used in the surgical procedure. The indication for proximal femoral osteotomy was determined from preoperative radiographic and CT-based assessment of proximal femoral morphology and the requirements for stable hip reduction. Varus and derotational correction was planned for patients with increased neck-shaft angle and femoral anteversion, taking into account dislocation severity and anticipated soft-tissue tension. The magnitude of correction was individualized according to patient-specific measurements and virtual surgical planning.

### 2.3 Surgical procedure

All procedures were performed by a single experienced surgeon under general anesthesia with the patient in the supine position. Open reduction and Salter pelvic osteotomy were performed first. Through a standard anterior approach, the iliac osteotomy was made from the sciatic notch to the anterior inferior iliac spine. A wedge-shaped bone graft was inserted into the osteotomy gap and fixed with two Kirschner wires. The femoral head was then reduced, and hip stability was reassessed in adduction and mild external rotation. Based on the preoperative plan and intraoperative assessment of hip stability, the target varus and derotational correction was confirmed before osteotomy. The correction was individualized to restore appropriate femoral alignment and stable concentric reduction, while excessive femoral retroversion was avoided to reduce the risk of posterior instability.

Proximal femoral osteotomy was subsequently performed through a standard lateral femoral approach in both groups with assistance from the modular surgical guide system. After exposure of the femoral shaft, the positioning guide was placed at the level of the lesser trochanter, and Kirschner wires were inserted according to the preoperative plan. The connecting guide was then mounted to guide distal wire placement. After removal of the connecting guide, the oblique and transverse osteotomy guides were sequentially installed, and the osteotomy was completed.

In the conventional group, fixation was achieved using a standard LCP-PHP of appropriate size according to the preoperative plan. In the customized group, fixation was achieved using the patient-specific plate. After the planned derotation and varus correction had been completed, centering sleeves matched to the Kirschner wires were inserted into the screw holes of the customized plate, and Kirschner wires were placed through the sleeves to assist positioning. The sleeves and wires were then removed sequentially, and definitive screw fixation was completed.

Because both groups underwent the same open reduction, Salter pelvic osteotomy, and modular guide-assisted proximal femoral osteotomy, the principal between-group difference was the fixation device used for the femoral osteotomy.

After surgery, the affected limb was immobilized using a single hip spica brace, maintaining the limb in 45° abduction and mild flexion with internal rotation. The brace was worn for 8–12 weeks until the osteotomy site had healed.

### 2.4 Observational indicators

Intraoperative data, including total blood loss, fluoroscopy frequency (covering both acetabular and femoral procedures), and the duration of the femoral procedure, were also documented. The duration of the femoral procedure was defined as the interval from femoral skin incision to confirmation of plate fixation by C-arm fluoroscopy.

Demographic and preoperative data, including age, sex, operated side, Tönnis grade, femoral NSA, FAA, acetabular index (AI), and center-edge angle (CE angle), were recorded for all patients. The absolute deviation from the planned correction was calculated for NSA and FAA and was defined as the absolute difference between the preoperatively planned angle and the corresponding early postoperative angle.

Follow-up data included final follow-up NSA, FAA, AI, CE angle, Severin classification, McKay score, and postoperative complications. Postoperative follow-up was performed according to a standardized institutional protocol. Patients were routinely evaluated at approximately 6 weeks, 3, 6, and 12 months after surgery, and annually thereafter until the final follow-up. During routine follow-up, clinical examination and standard anteroposterior pelvic radiographs were mainly used to evaluate osteotomy healing, hip stability, acetabular development, implant position, and implant-related complications. CT was not performed at each follow-up visit. Instead, CT examination with three-dimensional reconstruction was performed at the final follow-up to assess the final osseous morphology and femoral alignment. To reduce radiation exposure while maintaining image quality sufficient for bone segmentation and three-dimensional reconstruction, CT scanning parameters were adjusted according to the patient's age and body size, and the scanning range was limited to the affected hip and proximal femoral region. Radiographic evaluations at the final follow-up included measurement of NSA, FAA, AI, and CE angle, as well as assessment according to the Severin classification. Hip function was evaluated using the McKay scoring system. Complications, including infection, redislocation, avascular necrosis of the femoral head, implant migration, implant breakage, and nonunion, were recorded throughout the follow-up period.

Clinical evaluations and radiographic assessments were performed by orthopedic surgeons who were not involved in the implant design process. Quantitative radiographic measurements based on the final follow-up imaging data were independently performed by three observers who were blinded to group allocation and clinical outcomes. Each observer repeated all measurements twice, once in the first week (W1) and once in the second week (W2), with a 1-week interval between the two measurement sessions to minimize recall bias. For the main outcome analyses, the final representative value of each parameter was used.

### 2.6 Data analyses

All statistical analyses were performed using SPSS version 27.0 (IBM Corp., Armonk, NY, USA). Categorical variables were presented as frequencies or percentages and compared using the chi-square test. Continuous variables were expressed as the mean ± standard deviation (SD). The normality of the data distribution was assessed using the Shapiro–Wilk test. For normally distributed variables, intergroup comparisons were performed using the independent-samples t-test; otherwise, the Mann–Whitney U test was used. A *P* value < 0.05 was considered statistically significant.

For radiographic reliability analysis, inter-observer and intra-observer agreement were assessed for NSA, FAA, AI, and CE angle. Inter-observer reliability was evaluated separately at W1 and W2 using the measurements of the three observers, where W1 and W2 represented the first and second weeks of repeated measurements, respectively. Intra-observer reliability was assessed by comparing repeated measurements at W1 and W2 for each observer. Intraclass correlation coefficients (ICCs) were calculated using a two-way random-effects model with absolute agreement, and single-measure ICCs were reported. ICC values were interpreted as follows: < 0.50, poor; 0.50–0.75, moderate; 0.75–0.90, good; and > 0.90, excellent reliability (12).

The standard error of measurement (SEM) was calculated using the formula SEM = SD × √(1 – ICC), where SD was the standard deviation of the final representative value for each parameter and ICC was the intraclass correlation coefficient for intra-observer reliability. The minimal detectable change at the 95% confidence level (MDC95) was calculated as MDC95 = 1.96 × √2 × SEM (17). Bland–Altman analysis was additionally performed for NSA to assess systematic bias and agreement between repeated measurements. For each patient, the mean of the three observers' W1 measurements and the mean of the three observers' W2 measurements were calculated, and the difference was defined as W1 – W2 (18).

## 3 Results

A total of 40 patients were included in this study, including 18 in the conventional group and 22 in the customized group. The mean follow-up duration was 32.32 ± 4.18 months in the customized plate group and 31.39 ± 3.88 months in the conventional LCP-PHP group. All surgical incisions healed by primary intention, and satisfactory union was achieved at both the pelvic and femoral osteotomy sites in all patients. During follow-up, no complications such as infection, redislocation, avascular necrosis of the femoral head, implant migration, or implant breakage were observed in either group.

### 3.1 Demographic and preoperative data

The demographic characteristics and preoperative radiographic parameters of the two groups are summarized in Table 1. No significant differences were observed between the customized and conventional groups in age (*P* = 0.256), sex distribution (*P* = 0.972), Tönnis grade (*P* = 0.922), side of involvement (*P* = 0.842), or follow-up duration (*P* = 0.475). Similarly, there were no significant between-group differences in preoperative NSA (*P* = 0.624), FAA (*P* = 0.873), AI (*P* = 0.934), or CE angle (*P* = 0.180), indicating that the baseline characteristics were comparable between the two groups.

**Table 1:** Comparison of demographic data and preoperative parameters between the customized plate group and conventional plate group.

Indicators	Customized group (n = 22)	Conventional group (*n* = 18)	Statistics	P value
Age (years)	4.27 ± 1.08	3.89 ± 1.02	t=1.147	0.256
Gender				0.972
Male	6 (27.3)	5 (27.8)		
Female	16 (72.7)	13 (72.2)		
Side				0.842
Right	8 (36.4)	6 (33.3)		
Left	14 (63.6)	12 (66.7)		
Tönnis grade				0.922
II	5 (22.7)	5 (27.8)		
III	14 (63.6)	11 (61.1)		
IV	3 (13.6)	2 (11.1)		
Preoperative neck-shaft angle (°)	147.18 ± 2.91	147.61 ± 2.50	t=-0.494	0.624
Preoperative anteversion angle (°)	44.34 ± 3.01	44.5 ± 3.24	t=-0.161	0.873
Preoperative acetabular index (°)	35.5 ± 1.87	35.56 ± 2.33	t=-0.084	0.934
Preoperative CE angle (°)	12.36 ± 1.14	13.94 ± 1.55	t=-1.37	0.18
Follow-up time (months)	32.32 ± 4.18	31.39 ± 3.88	t=0.722	0.475

NSA, neck-shaft angle; FAA, femoral anteversion angle; AI, acetabular index; CE angle, center-edge angle.

### 3.2 Intraoperative outcomes and postoperative radiographic assessment

The intraoperative outcomes and immediate postoperative radiographic results are presented in Table 2. All procedures were successfully completed in both groups.

**Table 2:** Comparison of intraoperative data and postoperative radiographic angles between the customized plate group and conventional plate group.

Indicators	Customized group (*n* = 22)	Conventional group (*n* = 18)	Statistics	*P* value
Blood (ml)	277.05 ± 42.11	283.33 ± 37.65	*t* = 0.492	0.625
Time (hours)	0.43 ± 0.05	0.44 ± 0.04	*t* = 0.827	0.413
X-ray fluoroscopy times, times	2.86 ± 0.35	3 ± 0.49	*t* = 1.03	0.309
Postoperative neck-shaft angle (°)	110.86 ± 1.36	111.22 ± 1.26	*t* = −0.858	0.396
Postoperative anteversion angle (°)	21.18 ± 1.37	21.44 ± 1.04	*t* = −0.67	0.507
Postoperative acetabular index (°)	23.27 ± 1.28	22.56 ± 1.50	*t* = 1.63	0.111
Postoperative CE angle (°)	31.00 ± 1.35	31.72 ± 0.96	*t* = −1.91	0.063
NSA loss (°)	1.03 ± 0.2	3.26 ± 0.26	*t* = −30.588	< 0.001
FAA loss (°)	1.39 ± 0.15	1.48 ± 0.17	*t* = −1.846	0.073

NSA, neck-shaft angle; FAA, femoral anteversion angle; AI, acetabular index; CE angle, center-edge angle.

No significant differences were observed between the customized and conventional groups in intraoperative blood loss (277.05 ± 42.11 mL vs. 283.33 ± 37.65 mL, *P* = 0.625), operative time for the femoral procedure (0.43 ± 0.05 h vs. 0.44 ± 0.04 h, P = 0.413), or intraoperative fluoroscopic examinations (2.86 ± 0.35 vs. 3.00 ± 0.49, *P* = 0.309).

With regard to postoperative radiographic parameters, no significant between-group differences were found in postoperative NSA (110.86 ± 1.36° vs. 111.22 ± 1.26°, *P* = 0.396), FAA (21.18 ± 1.37° vs. 21.44 ± 1.04°, *P* = 0.507), AI (23.27 ± 1.28° vs. 22.56 ± 1.50°, *P* = 0.111), or CE angle (31.00 ± 1.35° vs. 31.72 ± 0.96°, *P* = 0.063).

The absolute deviation from the planned NSA correction was smaller in the customized group than in the conventional group (1.03 ± 0.20° vs. 3.26 ± 0.26°, *P* < 0.001), indicating closer agreement between the planned and achieved NSA. The corresponding deviation for FAA was comparable between groups (1.39 ± 0.15° vs. 1.48 ± 0.17°, *P* = 0.073).

### 3.3 Clinical and radiographic outcomes at final follow-up

The clinical and radiographic outcomes at final follow-up are summarized in Table 3. At the final follow-up, no significant differences were found between the customized and conventional groups in femoral NSA (111.00 ± 1.27° vs. 111.50 ± 1.25°, *P* = 0.220), FAA (21.36 ± 1.22° vs. 21.61 ± 0.91°, *P* = 0.480), AI (23.50 ± 0.96° vs. 22.89 ± 1.23°, *P* = 0.086), or CE angle (30.68 ± 2.50° vs. 31.83 ± 0.86°, *P* = 0.070).

**Table 3:** Comparison of clinical and radiographic outcomes at final follow-up between the customized plate group and conventional plate group.

Indicators	Customized group (*n* = 22)	Conventional group (n=18)	Statistics	P value
Final follow-up neck-shaft angle (°)	111.00 ± 1.27	111.5 ± 1.25	*t* = −1.247	0.22
Final follow-up anteversion angle (°)	21.36 ± 1.22	21.61 ± 0.91	*t* = −0.713	0.48
Final follow-up acetabular index (°)	23.5 ± 0.96	22.89 ± 1.23	*t* = 1.762	0.086
Final follow-up CE angle (°)	30.68 ± 2.50	31.83 ± 0.86	*t* = −1.87	0.07
Mckay			0.755	
Excellent	19 (86.4)	14 (77.8)		
Good	2 (9.1)	3 (16.7)		
Fair	1 (4.5)	1 (5.6)		
Poor	0	0		
Severin			0.471	
Excellent	20 (90.9)	15 (83.3)		
Good	2 (9.1)	3 (16.7)		
Fair	0	0		
Poor	0	0		

NSA, neck-shaft angle; FAA, femoral anteversion angle; AI, acetabular index; CE angle, center-edge angle.

Hip function was evaluated using the McKay criteria. In the customized group, the excellent, good, fair, and poor results were 19, 2, 1, and 0 cases, respectively, corresponding to an excellent-to-good rate of 95.5%. In the conventional group, the corresponding numbers were 14, 3, 1, and 0 cases, with an excellent-to-good rate of 94.4%. The difference between the two groups was not statistically significant (*P* = 0.755).

Radiographic outcomes were assessed according to the Severin classification. In the customized group, 20 hips were rated as excellent and 2 as good, whereas in the conventional group, 15 hips were rated as excellent and 3 as good. The excellent-to-good rate was 100% in both groups, and no significant difference was found between the two groups (*P* = 0.471).

### 3.4 Reliability of radiographic measurements

**Figure 8:**
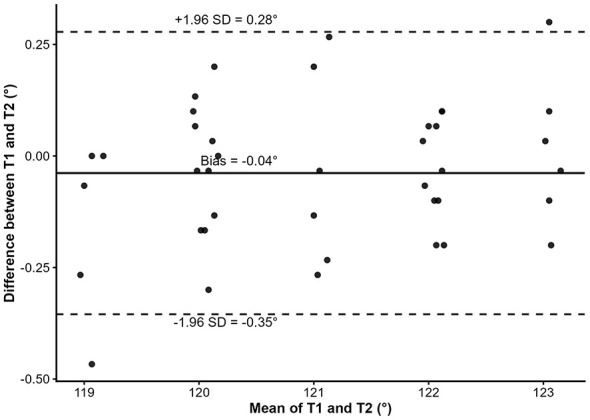
Bland–Altman plot for repeated NSA measurements. The solid line represents the mean bias, and the dashed lines represent the 95% limits of agreement. The mean bias was −0.04°, with upper and lower limits of agreement of 0.28° and −0.35°, respectively. NSA: neck-shaft angle.

Detailed reliability and measurement error results are summarized in Table 4, and Bland–Altman analysis for NSA is shown in Figure 8. The reliability analysis demonstrated excellent reproducibility of all radiographic measurements. For inter-observer reliability, the ICCs at W1 were 0.975 for NSA, 0.956 for FAA, 0.943 for AI, and 0.949 for the CE angle, while the corresponding ICCs at W2 were 0.973, 0.949, 0.949, and 0.944, respectively. Intra-observer reliability was also excellent. For NSA, the ICCs of the three observers were 0.971, 0.981, and 0.983, with a mean intra-observer ICC of 0.978. For FAA, the ICCs were 0.967, 0.974, and 0.964, with a mean of 0.968. For AI, the ICCs were 0.959, 0.943, and 0.967, with a mean of 0.956. For the CE angle, the ICCs were 0.973, 0.941, and 0.963, with a mean of 0.959.

**Table 4:** Reliability and measurement error of radiographic measurements.

Parameter	Inter-observer ICC at W1	Inter-observer ICC at W2	Mean intra-observer ICC	SEM (°)	MDC95 (°)
NSA	0.975	0.973	0.978	0.19	0.53
FAA	0.956	0.949	0.968	0.22	0.6
AI	0.943	0.949	0.956	0.3	0.82
CE angle	0.949	0.944	0.959	0.25	0.69

ICC, intraclass correlation coefficient; SEM, standard error of measurement; MDC95, minimal detectable change at the 95% confidence level; NSA, neck-shaft angle; FAA, femoral anteversion angle; AI, acetabular index; CE angle, center-edge angle.

The corresponding SEM values were 0.19° for NSA, 0.22° for FAA, 0.30° for AI, and 0.25° for the CE angle. The MDC95 values were 0.53°, 0.60°, 0.82°, and 0.69°, respectively. Bland–Altman analysis for NSA showed a mean bias of −0.04°, with 95% limits of agreement ranging from −0.35° to 0.28°, indicating good repeatability and limited measurement error.

## 4 Discussion

3D printing technology has been increasingly applied in orthopedic surgery, particularly in the management of complex deformities, osteotomies, and fracture reconstruction (19, 20). However, the application of patient-specific fixation implants in proximal femoral osteotomy for children with DDH has rarely been reported. To the best of our knowledge, this study represents one of the first attempts to apply a patient-specific proximal femoral plate in this clinical setting. The principal finding of the present study was that, compared with the conventional LCP-PHP, the customized plate resulted in a significantly smaller loss of the planned NSA correction.

When interpreting this finding, baseline comparability between the two groups should first be considered. In the present study, no significant differences were observed in demographic and clinical variables, including age, sex, side of involvement, follow-up duration, and Tönnis grade distribution. Preoperative radiographic parameters, including NSA, FAA, AI, and CE angle, were also comparable between groups. These findings minimized the possibility that the smaller NSA correction loss observed in the customized plate group was primarily attributable to baseline imbalance between the two groups.

Maintenance of NSA after proximal femoral osteotomy is particularly important because it directly influences hip congruency, load distribution across the femoral head, and the biomechanical environment required for subsequent acetabular remodeling (21–23). A possible explanation for this finding lies in the difference in plate–bone conformity between the two fixation methods. In conventional LCP-PHP fixation, the proximal segment of the plate and the proximal femur may show a slight angular mismatch. During tightening of the proximal locking screws, the engagement between the screw head and the plate thread may pull the cortical bone toward the plate, potentially causing subtle intraoperative loss of the intended varus correction. In contrast, the patient-specific plate used in this study was designed on the basis of a simulated post-osteotomy femoral model and achieved precise face-to-face contact with the bone surface. This improved conformity may reduce micromotion of the proximal segment during screw insertion and tightening, thereby better preserving the planned coronal-plane correction. This mechanism likely explains the superior maintenance of NSA observed in our cohort.

Because preoperative DDH severity may influence surgical difficulty, postoperative stability, and radiographic remodeling, we further compared the distribution of Tönnis grades between the two groups. No significant difference was observed, suggesting that the baseline radiographic severity of DDH was generally comparable between the customized plate group and the conventional LCP-PHP group. Therefore, the smaller NSA correction loss observed in the customized plate group was unlikely to be mainly attributable to an imbalance in preoperative DDH severity.

Importantly, the reliability analysis further strengthens the validity of this finding. Although the absolute angular difference between groups may appear numerically small, the radiographic measurements in this study showed excellent reproducibility. For NSA, both inter-observer and intra-observer ICCs were high, while the SEM was low (0.19°) and the MDC95 was only 0.53°. In addition, Bland–Altman analysis demonstrated minimal bias (−0.04°) and narrow 95% limits of agreement (−0.35° to 0.28°), indicating limited measurement error and good repeatability. Therefore, the between-group difference in NSA correction loss is unlikely to be attributable solely to measurement inconsistency. Notably, the absolute difference in NSA correction loss between the two groups (approximately 2.23°) was substantially greater than the MDC95, further supporting that this difference reflects a true effect rather than random measurement variation.

In contrast to NSA, no significant between-group difference was observed for FAA. This discrepancy may be related to the characteristics of the modular osteotomy technique used in the present study. Unlike traditional point-to-surface osteotomy configurations, the modular guide system creates a face-to-face osteotomy interface between the proximal and distal segments. Once the intended rotational correction has been achieved, this configuration provides a larger contact area and greater intrinsic stability, thereby allowing FAA to be maintained with less dependence on plate fixation. This may explain why the advantage of the customized plate was more evident in maintaining coronal alignment than in rotational alignment.

Because both groups underwent the same osteotomy procedure and differed mainly in the fixation implant applied after correction, marked differences in intraoperative blood loss, operative time, and fluoroscopy frequency were not expected. Accordingly, although the customized group showed numerically lower blood loss, shorter operative time, and fewer fluoroscopic examinations, these differences did not reach statistical significance. Therefore, the clinical relevance of the present study lies primarily in the ability of the customized plate to better maintain the planned correction, rather than in broad perioperative efficiency gains.

At the final follow-up, functional outcomes and radiographic classifications were comparable between the two groups. Likewise, radiographic parameters, including NSA, FAA, AI, and CE angle, showed no significant intergroup differences either immediately postoperatively or at the final follow-up. Moreover, no major complications were observed in either group, such as infection, redislocation, avascular necrosis of the femoral head, implant migration, or implant breakage. Previous studies have shown that the LCP-PHP is a safe and effective fixation option for proximal femoral osteotomy in children with DDH (13, 24, 25). The findings of the present study not only confirm the favorable clinical outcomes reported in earlier studies, but also extend the existing evidence by suggesting that the patient-specific customized plate is likewise a feasible and safe option for pediatric proximal femoral osteotomy. Taken together, these results indicate that the main benefit of the customized plate may be improved maintenance of the planned correction rather than superior short-term clinical or radiographic outcomes.

From a broader perspective, the combination of the modular osteotomy technique and the patient-specific customized plate may be understood as an integrated digital workflow linking preoperative planning, osteotomy execution, and definitive fixation. The modular guide system enables accurate three-dimensional correction and produces a stable face-to-face osteotomy interface, while the customized plate further optimizes plate–bone conformity after correction. This combination may help preserve consistency between the preoperative surgical plan and the final reconstructed femoral alignment, thereby improving the overall accuracy and stability of reconstruction. The present study provides preliminary evidence that this integrated workflow is feasible and safe and can improve reproduction of the planned NSA correction. Its clinical implementation requires additional CT-based planning, engineering support, manufacturing time, and financial resources. Given the absence of superior short-term functional or radiographic outcomes, the current evidence does not support routine application in all children undergoing proximal femoral osteotomy. The technique may be more relevant for selected patients with complex proximal femoral anatomy or substantial rotational deformity, in whom accurate transfer of the preoperative plan is particularly important. Further studies incorporating economic outcomes, preparation time, radiation exposure, and long-term clinical benefits are needed to define its practical and cost-effective use.

Several limitations of the present study should also be acknowledged. First, this was a retrospective study conducted at a single center, which may have introduced selection bias. Second, the relatively small sample size may have limited the statistical power of the analysis, particularly with respect to functional outcomes, radiographic classifications, and uncommon complications. Third, repeated CT examinations increased cumulative radiation exposure in this pediatric population, although age- and body-size–adjusted scanning parameters and a restricted scanning range were used. The cumulative radiation dose was not quantitatively recorded or analyzed. Future studies should evaluate lower-dose protocols and alternative three-dimensional imaging methods. Fourth, manufacturing costs, preoperative preparation time, and resource utilization were not systematically recorded; therefore, the cost-effectiveness of the patient-specific workflow could not be evaluated. Fifth, the follow-up period was relatively short and may not fully reflect long-term acetabular development or functional outcomes. Future studies with larger sample sizes, prospective designs, and longer follow-up periods are needed to further validate the clinical value of this technique.

## 5 Conclusion

In conclusion, the combination of a modular 3D-printed osteotomy guide system and a patient-specific customized proximal femoral plate represents a feasible and safe fixation strategy for proximal femoral osteotomy in children with developmental dysplasia of the hip. Compared with the conventional LCP-PHP, the customized plate more accurately maintained the planned neck-shaft angle correction. Although short-term radiographic and functional outcomes were comparable between groups, this integrated digital workflow may offer a promising approach for improving correction accuracy in pediatric hip reconstruction.

## Data Availability

The raw data supporting the conclusions of this article will be made available by the authors, without undue reservation.
